# Genomic profiling and network-level understanding uncover the potential genes and the pathways in hepatocellular carcinoma

**DOI:** 10.3389/fgene.2022.880440

**Published:** 2022-11-21

**Authors:** Sherif A. El-Kafrawy, Mai M. El-Daly, Leena H. Bajrai, Thamir A. Alandijany, Arwa A. Faizo, Mohammad Mobashir, Sunbul S. Ahmed, Sarfraz Ahmed, Shoaib Alam, Raja Jeet, Mohammad Amjad Kamal, Syed Tauqeer Anwer, Bushra Khan, Manal Tashkandi, Moshahid A. Rizvi, Esam Ibraheem Azhar

**Affiliations:** ^1^ Special Infectious Agents Unit-BSL3, King Fahd Medical Research Centre, King Abdulaziz University, Jeddah, Saudi Arabia; ^2^ Department of Medical Laboratory Sciences, Faculty of Applied Medical Sciences, King Abdulaziz University, Jeddah, Saudi Arabia; ^3^ Biochemistry Department, Faculty of Sciences, King Abdulaziz University, Jeddah, Saudi Arabia; ^4^ Department of Microbiology, Tumor and Cell Biology (MTC), Karolinska Institute, Stockholm, Sweden; ^5^ Genome Biology Lab, Department of Biosciences, Jamia Millia Islamia, New Delhi, India; ^6^ Department of Biosciences, Jamia Millia Islamia, New Delhi, India; ^7^ Department of Biotechnology, Jamia Millia Islamia, New Delhi, India; ^8^ Botany Department, Ganesh Dutt College, Begusarai, Bihar, India; ^9^ Institutes for Systems Genetics, Frontiers Science Center for Disease-related Molecular Network, West China Hospital, Sichuan University, Chengdu, China; ^10^ King Fahd Medical Research Center, King Abdulaziz University, Jeddah, Saudi Arabia; ^11^ Department of Pharmacy, Faculty of Allied Health Sciences, Daffodil International University, Dhaka, Bangladesh; ^12^ Enzymoics, Hebersham, NSW, Australia; ^13^ Novel Global Community Educational Foundation, Hebersham, NSW, Australia; ^14^ Department of Biochemistry, College of Science, University of Jeddah, Jeddah, Saudi Arabia

**Keywords:** HCV and HCC, biomarkers, gene expression/mutational profiling, co-expression, network-level understanding

## Abstract

Data integration with phenotypes such as gene expression, pathways or function, and protein-protein interactions data has proven to be a highly promising technique for improving human complex diseases, particularly cancer patient outcome prediction. Hepatocellular carcinoma is one of the most prevalent cancers, and the most common cause is chronic HBV and HCV infection, which is linked to the majority of cases, and HBV and HCV play a role in multistep carcinogenesis progression. We examined the list of known hepatocellular carcinoma biomarkers with the publicly available expression profile dataset of hepatocellular carcinoma infected with HCV from day 1 to day 10 in this study. The study covers an overexpression pattern for the selected biomarkers in clinical hepatocellular carcinoma patients, a combined investigation of these biomarkers with the gathered temporal dataset, temporal expression profiling changes, and temporal pathway enrichment following HCV infection. Following a temporal analysis, it was discovered that the early stages of HCV infection tend to be more harmful in terms of expression shifting patterns, and that there is no significant change after that, followed by a set of genes that are consistently altered. PI3K, cAMP, TGF, TNF, Rap1, NF-kB, Apoptosis, Longevity regulating pathway, signaling pathways regulating pluripotency of stem cells, Cytokine-cytokine receptor interaction, p53 signaling, Wnt signaling, Toll-like receptor signaling, and Hippo signaling pathways are just a few of the most commonly enriched pathways. The majority of these pathways are well-known for their roles in the immune system, infection and inflammation, and human illnesses like cancer. We also find that ADCY8, MYC, PTK2, CTNNB1, TP53, RB1, PRKCA, TCF7L2, PAK1, ITPR2, CYP3A4, UGT1A6, GCK, and FGFR2/3 appear to be among the prominent genes based on the networks of genes and pathways based on the copy number alterations, mutations, and structural variants study.

## Introduction

Acquired genomic aberrations of various sorts and sizes, ranging from single nucleotide variants to structural abnormalities, are a common feature of cancer. Cancer genomes have a wide range of genomic abnormalities of various sorts and sizes. Single nucleotide variants (SNVs) to bigger structural variants (SVs) all have an impact on genome organization (Bruin et al., 2013; Dienstmann et al., 2014; Prandi et al., 2014). Different types of mutations are seen in cancer cells, and they are linked to the cell’s ability to reproduce uncontrollably. Certain modifications to the genetic code only affect one or a few letters (Futreal et al., 2004; Zarrei et al., 2015; Yizhak et al., 2019). Others, referred to as copy number changes (CNA), involve bigger segments of the genome that can be deleted (deletions) or duplicated (duplications) (amplifications) (Pelham et al., 2006; Grubor et al., 2009; Agell et al., 2012; Li and Li, 2014). Various patients’ tumors have different quantities of these deletions or amplifications, which are collectively known as the CNAs burden. Scientists can now scan the genomes of cancers and assess the types of mutations present in each patient thanks to new technologies. The outcomes can assist in determining the best course of action. Patients with a high CNAs burden in their tumors, for example, have a higher chance of relapse after treatment. However, it is unclear whether these persons have shorter survival rates as well, or whether CNAs levels might predict the prognosis of other cancers. Over a hundred samples from prostate cancer patients who were not treated with surgery or radiation were analyzed by Hieronymus et al. The findings revealed that a higher CNA burden in tumors is linked to more disease-related mortality (Rigaill et al., 2012; Beerenwinkel et al., 2014; Li and Li, 2014; Cooper et al., 2015). The findings in prostate cancer were also true in other cancer types. When Hieronymus et al. looked at genomic data from individuals with various tumors using a different DNA sequencing assay that is authorized for clinical use, they came to the same conclusions. This suggests that CNA load could be a valuable clinical measure for assessing risk in cancer patients. Structural variation, in which rearrangements remove, increase, or reorganize genomic regions ranging in size from kilobases (kb) to whole chromosomes, is a crucial mutational mechanism in cancer. Somatically acquired big structural variations (SVs) are a type of abnormality that can cause cancer by deactivating tumor suppressor genes and upregulating oncogenes, among other things. Detecting and characterizing these variations could lead to better cancer medicines and diagnostics (Lim and van Oudenaarden, 2007; Barbosa-Morais et al., 2010; Biesecker and Spinner, 2013; Gerstberger et al., 2014; Moncunill et al., 2014; Zarrei et al., 2015).

Cancer is caused by beginning cells that undergo a lot of evolutionary selection as the disease progresses and can change dramatically throughout treatment. Tumor cell evolution may result in subclonal divergence, leading in genetic and molecular heterogeneity. Computational approaches for creating maps of cancer evolution could help clinical risk classification and therapy techniques. There is still a gap in the study of slightly aberrant or extremely varied malignancies, despite the development of tools for assessing tumor DNA purity and cancer cell ploidy (Bardwell et al., 2001; Thomas et al., 2004; Cui et al., 2007; Carja and Feldman, 2012; Klinke, 2013; Murugaesu et al., 2013; Paguirigan et al., 2015).

The most common type of cancer in the world, hepatocellular carcinoma (HCC), is the leading cause of cancer-related fatalities (Ieta et al., 2007; Consortium et al., 2010; El-Serag, 2011; Repana and Ross, 2015; HASS et al., 2016). A high number of HCC patients show signs of vascular invasion with intrahepatic metastases, which tend to invade portal vein branches and create portal vein tumor thrombus (PVTT), which can obstruct the portal vein and cause portal hypertension (ROBINSON, 1994; Jhunjhunwala et al., 2014; Llovet et al., 2015). HCC advancement can be linked to a variety of causes, the most common of which being HBV and HCV. Aflatoxin B1, alcohol consumption, cigarette smoking, hepatotoxic chemical agents, and host co-factors such as elevated serum androgen levels, genetic polymorphisms, and DNA repair enzymes may all be linked to the progressive accumulation of a number of genomic aberrations within the hepatocytes, with TP53 and CTNNB1 being two well-known cancer drivers (Fujikawa et al., 2001; Ichikawa et al., 2008; Attari et al., 2019).

HCV is a single-stranded RNA virus with four structural proteins: capsid protein C, envelope glycoproteins E1 and E2, and protein P7, as well as six non-structural proteins: NS2, NS3, NS4A, NS4B, NS5A, and NS5B. Chronic inflammation, immune-mediated hepatocyte death and disorder, fibrosis, and multilayer diseases (cellular pathways such as proliferation, apoptosis, and DNA repair) are all possible outcomes of HCV infection (core and structural proteins) (Ahmad et al., 2012; Jost and Altfeld, 2013; Roberts and Gordenin, 2014; Schwarzenbach et al., 2014; Sacerdote and Ricceri, 2018; Lupberger et al., 2019b).

As previously noted, HCV infection appears to be a potential cause of liver disorders such as liver cancer, steatosis, and fibrosis, and the mechanisms behind infection, liver disease development, and carcinogenesis are not fully or well understood. There are also a number of factors associated with HCC. So, in order to learn more about the leading cause of liver cancer/hepatocellular carcinoma, we used a method in which we gathered and studied previously identified biomarkers, a publicly available dataset for hepatocellular carcinoma (temporal data) with and without HCV infection, a combined study, clinical relevance, and functional impact. We examined changes in gene expression patterns, mutation mutations, CNAs, and SVs using publically available information from Gene Expression Omnibus (GEO) and TCGA, followed by cBioPortal. Furthermore, we investigated the enriched pathways for their overall functional implications and used network-level understanding to determine the impact of changed genes on other genes.

## Results

As noted in the preceding section, we compiled a list of known HCC biomarkers before working with the GEO and TCGA datasets. The GEO dataset contains HCV-infected data that spans 10 days. So, in the first section of the results, we focused on data related to HCC biomarkers, followed by temporal gene expression profiling and functional significance, and finally, CNAs, mutations, and SVs analyses.

## HCC biomarkers and its clinical relevance

**FIGURE 1:**
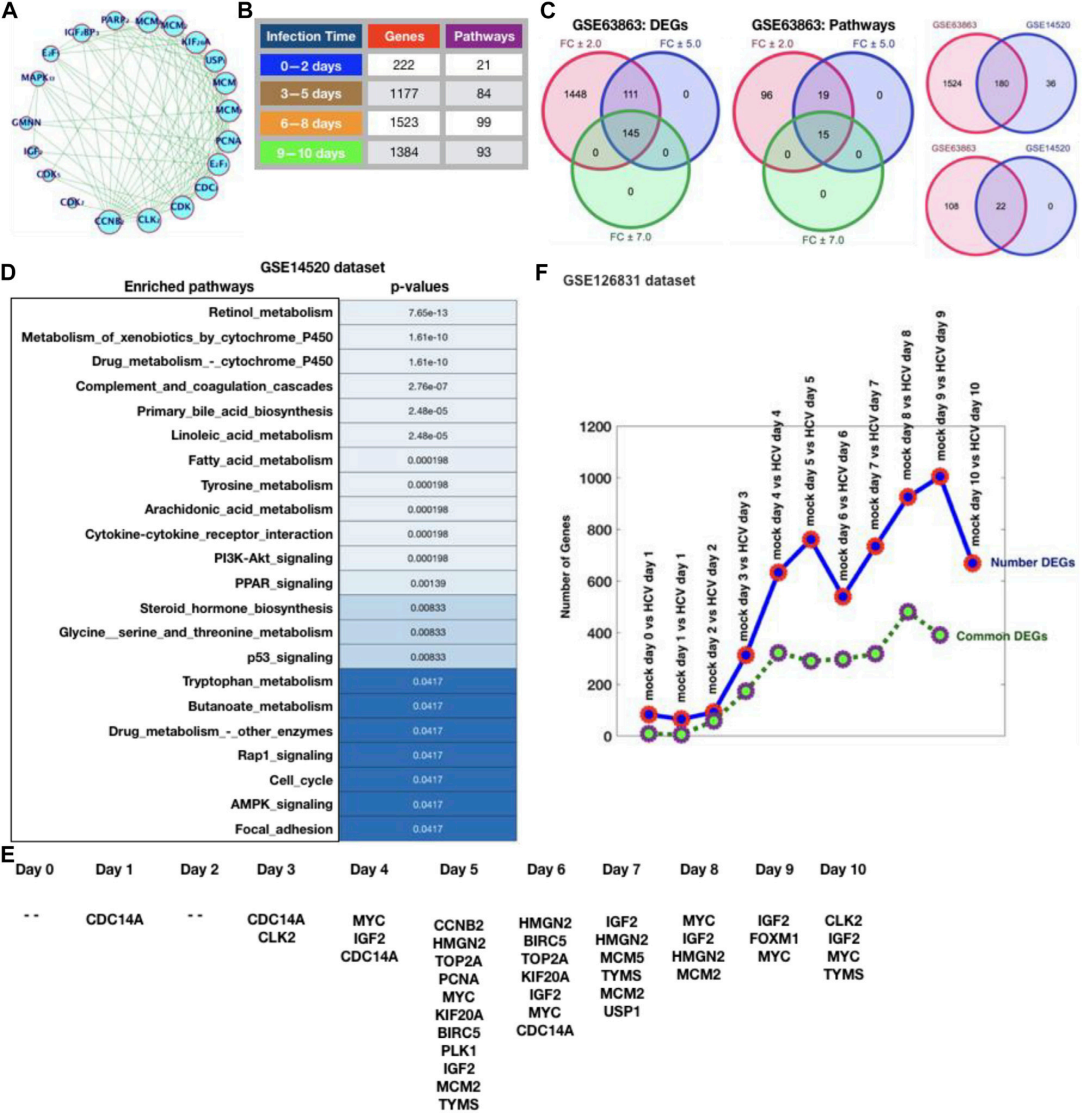
Differential gene expression profiling and pathway enrichment analysis. **(A)** Co-occurrence network. **(B)** Temporal evolution of gene expression aberrations and its functional consequences. **(C)** Venn diagram to represent the shared and specific genes and pathways which are potentially altered as a result of CRC. **(D)** Enriched pathways followed by their respective *p*-values. **(E)** Temporal gene expression profiling of HCC in result to HCV infection. The number of DEGs from day 1 to day 10 and number of common DEGs in different combinations (such day 1 with day 2, day 2 with day 3, day 3 with day 4, day 4 with day 5, and so on). **(F)** HCC biomarkers profiling for the temporal dataset.

Using cBioPortal in HCC, we were able to map out the proportion of over-expression (both individually and overall) and co-occurrence for the selected genes (biomarkers picked from previously published work) inside the TCGA database. We provided the co-occurrence in Figure 1A, and for co-occurrence, we also presented the network with the relevant connectivity in terms of co-occurrence. CCNB2, CLK2, CDK4, CDC7, E2F3, PCNA, MCM3, MCM4, USP1, KIF20A, MCM2, and MCM7 are shown to be dominantly controlling a large number of genes, or to put it another way, most of the genes are interdependent. The majority of the genes here are involved in the cell cycle, however there are a few that are specifically involved in infection and inflammatory processes (E2F5, MAPK13, IGF2BP3, IGF2). We investigated the temporal gene expression profiling for HCV infection acquired from GEO after assessing the biomarkers association. First, as shown in Figure 1B, we projected DEGs for each day of infection by combining the genes into four groups (0–2 days, 3–5 days, 6–8 days, and 9–10 days). Figure 1B shows that increased infection duration causes significant changes in gene expression patterns until a certain time point, after which there are few changes in gene expression patterns and a slight decrease in the number of DEGs between 9 and 10 days, as well as enriched pathways or biological functions affected by changes in gene expression patterns. Figure 1B shows an exponential growth in the number of DEGs up to day eight, after which there is volatility, leading to the conclusion that there is a greater level of distribution in gene expression pattern during early HCV infection in HCC.

PI3K, cAMP, TGF, TNF, Rap1, NF--kB, Apoptosis, Longevity regulating pathway, signaling pathways regulating pluripotency of stem cells, Cytokine-cytokine receptor interaction, p53 signaling, Wnt signaling, Toll-like receptor signaling, and Hippo signaling pathways are just a few of the most commonly enriched pathways. The majority of these pathways are well-known for their roles in the immune system, infection and inflammation, and human illnesses like cancer.

In addition, we conducted a comparison analysis of HCC gene expression datasets that were not infected with HCV. We observed that there are a large number of DEGs, so we prepared lists of DEGs for these three different fold changes, 2.0, 5.0, and 7.0, and analyzed the enriched pathways for all three datasets, finding that 145 DEGs and 15 enriched pathways were shared across all the three fold changes (2.0, 5.0, and 7.0), 111 DEGs and 19 enriched pathways shared between fold changes 2.0 and 5.0, and 1448 DEGs and 96 enriched pathways were unique to fold change 2.0. We compared this dataset to another dataset for the same after evaluating it at different fold changes. 180 DEGs and 22 enriched pathways were shared between the two datasets, and GSE63863 had its own set of DEGs and enriched pathways. The majority of these 22 pathways are well-known and acknowledged as the most important pathways linked to various malignancies, including HCC (Figures 1C,D; Supplementary Data S1). Furthermore, we have also presented the HCV-infected HCC temporal data in Figure 1E which contains temporal gene expression profiling of HCC in result to HCV infection. The number of DEGs from day 1 to day 10 and number of common DEGs in different combinations (such day 1 with day 2, day 2 with day 3, day 3 with day 4, day 4 with day 5, and so on).

Moreover, we have also performed the mapping of known HCC biomarkers with temporal gene expression dataset and observe that day 0 and day 2 have no HCC biomarkers as DEGs while day 5 contains the maximum number (11) of HCC biomarkers in the predicted DEGs list (Figure 1E).

## Analysis of CNAs, mutations, and SVs from TCGA database

**FIGURE 2:**
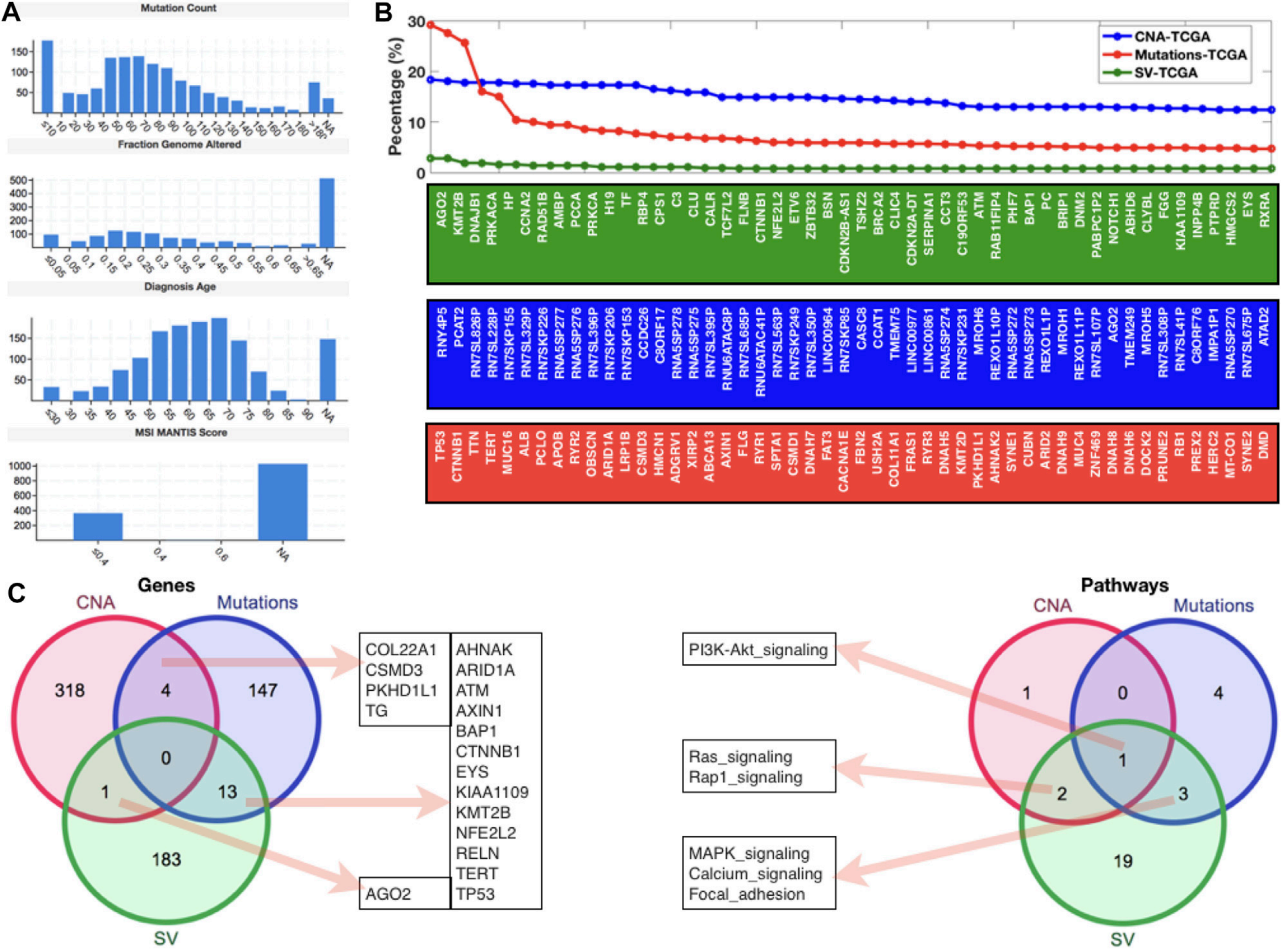
Genomic-level alterations in HCC datasets of TCGA database. **(A)** Histograms to present the mutation count, fraction genome altered, diagnosis age, and MSI mantis score. **(B)** Percentage of patients with different types of alterations (CNA, Mutations, and SV) in case of HCC. **(C)** Venn diagrams to display the shared and specific significant genes and pathways.

After examining gene expression profiling from the GEO database, we went on to look at global genomic aberrations using TCGA and cBioPortal, as well as all of the HCC datasets to look at overall CNAs, mutations, and SVs in the case of HCC. Figure 2A shows the MANTIS Score distributions for mutation count, fraction genome altered, diagnostic age, and microsatellite instability (MSI) (which predicts the MSI status of tumors). For this study, all of the HCC samples from TCGA were chosen. In terms of mutation count, we can see that 10 samples have the most (>150), while 40–70 samples have a similar number of mutations (>120 and 150), and the fraction of genome altered has similar histogram patterns. The majority of the diagnosed patients were between the ages of 50 and 75, with an MSI MANTIS score of 0.4 for almost 400 patients and an MSI MANTIS score of unknown for over 1000 samples. Figure 2B shows the top 50 genes after giving the fundamental data of mutations, CNAs, and SVs. Most of the top 50 genes are specific, although AGN2 (which plays a vital function in RNA interference) was found in both CNAs and SVs lists, and CTNNB1 (a putative component of the adherens junction) was found in both mutations and SVs lists. After mapping the top 50 genes, we applied a threshold level to all three scenarios (CNAs (10.0), mutations (3.0), and SVs (0.5)) and used a venn diagram to compare these gene lists to the enriched pathways lists (Figure 2C). We can see that none of these three lists have a gene in common. There were four genes shared by CNAs and the mutations list, thirteen genes shared by mutations and SVs, and one gene shared by SVs and the CNAs list. In terms of gene set comparison, one pathway (PI3K-AKT) was shared by all three lists, three pathways (MAPK, calcium, and focal adhesion signaling) were shared by mutations and SVs, and two routes (Ras and Rap1 signaling) were shared by SVs and CNAs (Figure 2C) (Table 1).

**TABLE 1:** Temporal enriched pathways.

Enriched pathways (CNA genes)	
KEGG_04151_PI3K-Akt_signaling_pathway_-_Homo_sapiens_(human)	1.984127e-04
KEGG_04015_Rap1_signaling_pathway_-_Homo_sapiens_(human)	8.333333e-03
KEGG_04014_Ras_signaling_pathway_-_Homo_sapiens_(human)	4.166667e-02
KEGG_04360_Axon_guidance	4.166667e-02
Enriched pathways (Mutated genes)	
KEGG_04151_PI3K-Akt_signaling_pathway_-_Homo_sapiens_(human)	2.480159e-05
KEGG_04510_Focal_adhesion	1.984127e-04
KEGG_04512_ECM-receptor_interaction	1.984127e-04
KEGG_04020_Calcium_signaling_pathway	8.333333e-03
KEGG_04010_MAPK_signaling_pathway	4.166667e-02
KEGG_04110_Cell_cycle	4.166667e-02
KEGG_04120_Ubiquitin_mediated_proteolysis	4.166667e-02
KEGG_04918_Thyroid_hormone_synthesis	4.166667e-02
Enriched pathways (SV genes)	
KEGG_04010_MAPK_signaling_pathway	2.755732e-06
KEGG_04610_Complement_and_coagulation_cascades	2.480159e-05
KEGG_04151_PI3K-Akt_signaling_pathway_-_Homo_sapiens_(human)	1.984127e-04
KEGG_04310_Wnt_signaling_pathway	1.984127e-04
KEGG_04510_Focal_adhesion	1.984127e-04
KEGG_00980_Metabolism_of_xenobiotics_by_cytochrome_P450	1.388889e-03
KEGG_04014_Ras_signaling_pathway_-_Homo_sapiens_(human)	1.388889e-03
KEGG_04611_Platelet_activation	1.388889e-03
KEGG_04810_Regulation_of_actin_cytoskeleton	1.388889e-03
KEGG_04919_Thyroid_hormone_signaling_pathway	1.388889e-03
KEGG_00830_Retinol_metabolism	8.333333e-03
KEGG_00982_Drug_metabolism_-_cytochrome_P450	8.333333e-03
KEGG_04020_Calcium_signaling_pathway	8.333333e-03
KEGG_04371_Apelin_signaling_pathway_-_Homo_sapiens_(human)	8.333333e-03
KEGG_04921_Oxytocin_signaling_pathway	8.333333e-03
KEGG_00020_Citrate_cycle_(TCA_cycle)	4.166667e-02
KEGG_04015_Rap1_signaling_pathway_-_Homo_sapiens_(human)	4.166667e-02
KEGG_04022_cGMP-PKG_signaling_pathway_-_Homo_sapiens_(human)	4.166667e-02
KEGG_04141_Protein_processing_in_endoplasmic_reticulum	4.166667e-02
KEGG_04144_Endocytosis	4.166667e-02
KEGG_04261_Adrenergic_signaling_in_cardiomyocytes	4.166667e-02
KEGG_04392_Hippo_Signaling_Pathway	4.166667e-02
KEGG_04550_Signaling_pathways_regulating_pluripotency_of_stem_cells	4.166667e-02
KEGG_04723_Retrograde_endocannabinoid_signaling	4.166667e-02
KEGG_04916_Melanogenesis	4.166667e-02

## Network-level understanding potential HCC genes

**FIGURE 3:**
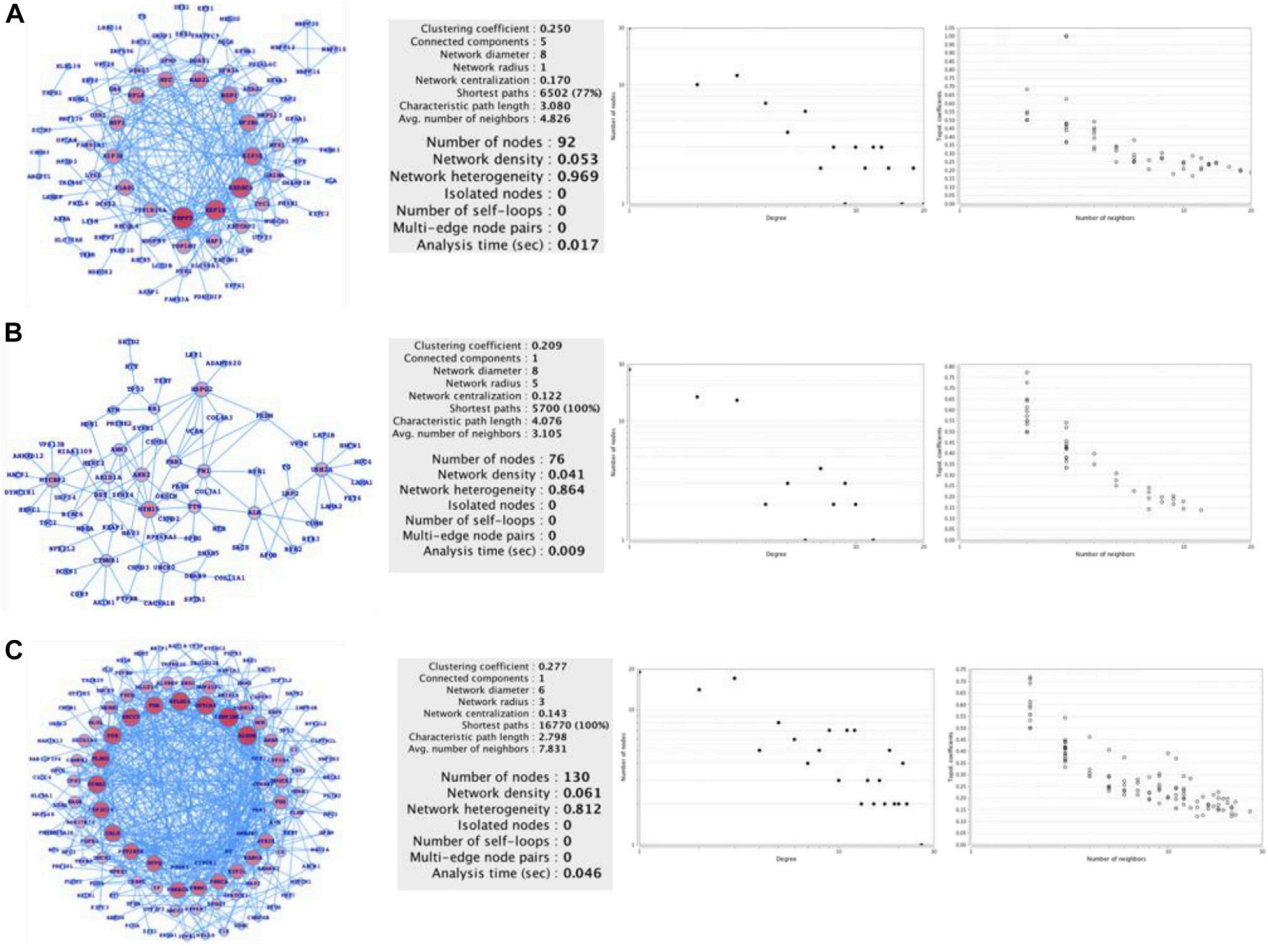
Network-level understanding top-ranked genes. **(A)** CNA genes network, **(B)** Mutated genes, and **(C)** SV genes network followed by their respective analysis (degree distribution and topological coefficients).

**FIGURE 4:**
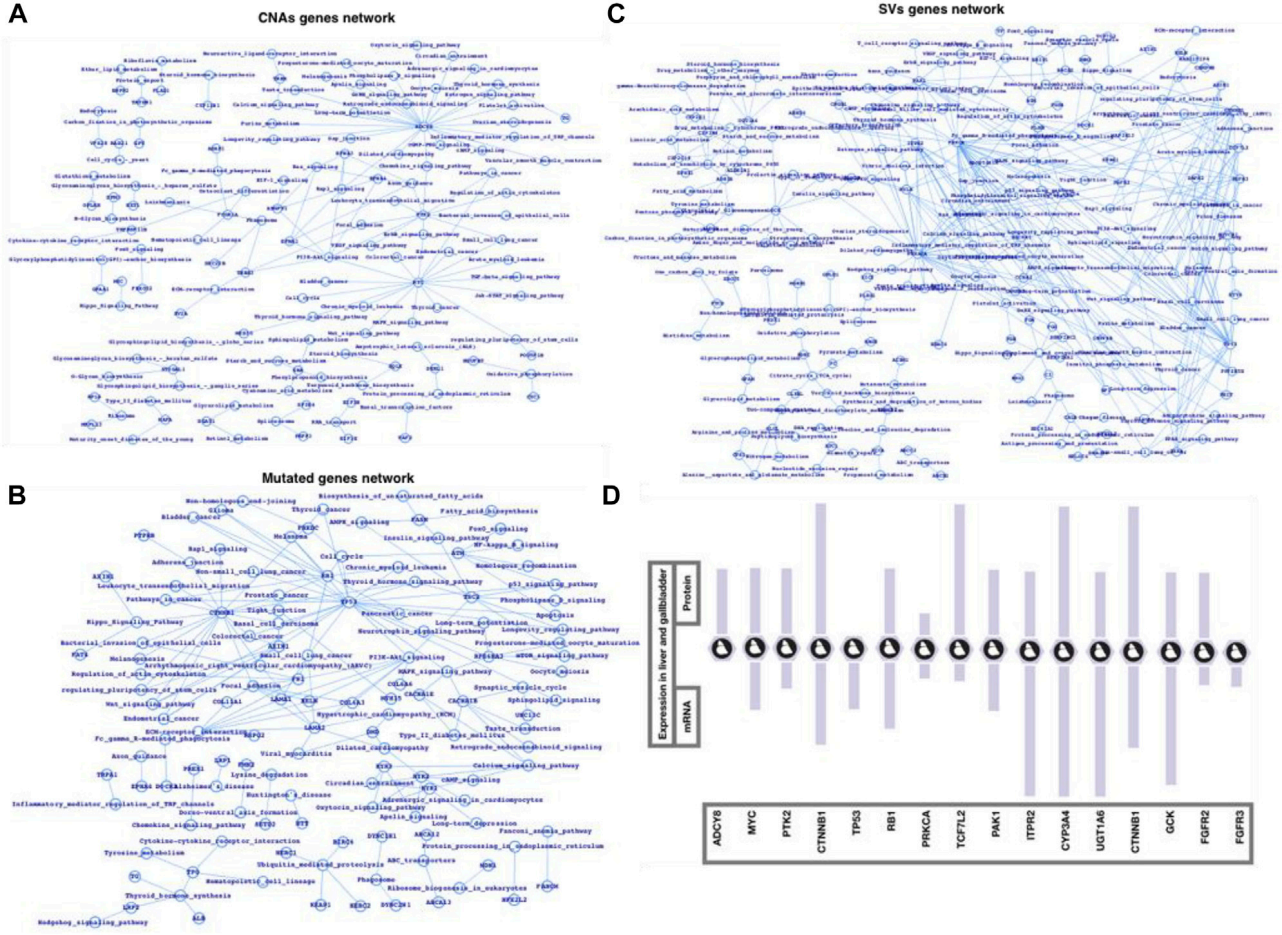
Network-level understanding top-ranked genes and the associated pathways. **(A)** CNA genes network, **(B)** Mutated genes, **(C)** SV genes network followed by their respective analysis, and **(D)** mRNA and protein expression in liver and gallbladder tissues (source protein atlas).

Finally, we used the FunCoup network database of CNAs, mutations, and SVs genes list to map out the networks, which we then processed in cytoscape using network analyzer (Figures 3A–C). The statistics, degree distribution, and topological coefficients of the networks were shown in Figure 3. The degree distribution, topological coefficients, and statistical features all show that the SVs network is densely connected, followed by the CNAs network and mutations network (thinly connected). PRPF3, EEF1D, EXOSC4, EIF3E, SF3B4, BOP1, RAD21, MYC, RPL8, HSF1, HIF3E, FLAD1, PPP1R16A, TOP1MT, MAF1, KRTCAP2, CYC1, and GRINA were shown to be substantially related in the CNAs genes network. MYH15, MYCBP2, HSPG2, USH2A, FN1, FBN1, CTNNB1, ARID1A, and TTN were shown to be substantially related in the mutant genes network. The strongly related genes in the SVs genes network were ALDOB, SERPINC1, UGT1A6, NPLOC4, FGA, KRCC5, FGB, PLRG1, CCNA2, CYP2C18, CALR, PPP2R5E, SFPQ, PRKACA, PBRM1, PRKCA, EIF3L, RAB6A, and STK38. Based on the general network notion, it might be concluded that genes that appear to be heavily connected within the network are more significant than genes that appear to be less connected. Similarly, the more coupled genes have the potential to change more genes, and as a result, more biological activities. Furthermore, we plotted the gene networks and associated pathways for CNAs genes, mutant genes, and SVs genes (Figures 4A–C), where ADCY8, MYC, and PTK2 appear to be part of a large number of essential signaling pathways in the case of the CNAs genes network. CTNNB1, TP53, and RB1 have all been linked to cancer or cancer-related signaling pathways, primarily in HCC. PRKCA, TP53, TCF7L2, PAK1, ITPR2, CYP3A4, UGT1A6, CTNNB1, GCK, and FGFR2/3 are among the genes in the SVs genes network that connect a vast number of signaling pathways. We conclude that the top-ranked CNAs, mutant, and SVs genes have the ability to change at a higher-scale at the functional level based on these three genes and pathways association networks.

## Discussion

Using GEO and TCGA datasets, we adopted an interdisciplinary strategy to investigate gene expression profiles, somatic mutations, CNAs, and SVs analyses. The gene expression datasets were divided into two categories: temporal datasets infected with HCV and non-temporal datasets clear of HCV infection. This study took into account all of the HCC datasets in the TCGA database. Furthermore, we used a network biology technique (Barabasi and Oltvai, 2004; Emmert-Streib and Glazko, 2010; Hu et al., 2016) to better understand the relationship between top-ranked genes in terms of linkage while they were altered. The SVs genes network appears to be the most densely connected, followed by the CNAs and mutant gene networks. Moreover, we have also used those data where the infection is associated with HBV to evaluate the broad spectrum of the impact of infection in addition to HCC at gene expression and functional levels.

The assessment of the clonality of each somatic aberration enables the deconvolution of the sequence of oncogenic events that occur during tumor initiation or progression. Assuming that clonal alterations originated prior to subclonal alterations within the same tumor, we examined pairs of genes that are aberrant in the same sample and across multiple tumors to determine the directionality of the clonal-subclonal hierarchy (Cibulskis et al., 2012; Klijn et al., 2013; Li and Li, 2014; Swanton, 2014). HCC subtypes are classified by gene clustering of tumor specific genes which resolve the HCC pathogenesis according to their etiological factor, clinical stage, recurrence rate, and prognosis. The expression in genes regulating cell proliferation and anti-apoptotic pathways such as PNCA and cell cycle regulators CDK4, CCNB1, CCNA2, and CKS2 and ubiquitination mechanisms were studied previously. In addition to that several molecular markers of tumor progression like HSP70, CAP2, GPC3, and GS were also expressed in expression profiling. The expression profiling by time course analysis has identified several genes as a progression marker in HCC such as GPC3, CXCL12, SPINK1, GLUL, UBD, TM4SF5, DPT, SCD, MAL2, TRIM55, and COL4A2. Meanwhile the specific alteration of HCC signals transduction pathways and protein expression have given the opportunities for new therapies targeting new molecular factors. High-throughput data (genomic and proteomic) are frequently generated with the goal to understand the genotype-phenotype relationship in the complex diseases (Emilsson et al., 2008; Gonzalez-Perez et al., 2013; van’t Veer et al., 2002).

Among the most common enriched pathways are PI3K, cAMP, TGF, TNF, Rap1, NF-kB, Apoptosis, Longevity regulating pathway, signaling pathways regulating pluripotency of stem cells, Cytokine-cytokine receptor interaction, p53 signaling, Wnt signaling, Toll-like receptor signaling, and Hippo signaling pathways. Majority of these pathways well characterized for immune controlling system, infection and inflammation, and human diseases such as cancer. PRPF3, EEF1D, EXOSC4, EIF3E, SF3B4, BOP1, RAD21, MYC, RPL8, HSF1, HIF3E, FLAD1, PPP1R16A, TOP1MT, MAF1, KRTCAP2, CYC1, and GRINA were highly connected in case of CNAs network, in mutated genes network, MYH15, MYCBP2, HSPG2, USH2A, FN1, FBN1, CTNNB1, ARID1A, and TTN were highly connected, and ALDOB, SERPINC1, UGT1A6, NPLOC4, FGA, KRCC5, FGB, PLRG1, CCNA2, CYP2C18, CALR, PPP2R5E, SFPQ, PRKACA, PBRM1, PRKCA, EIF3L, RAB6A, and STK38 were among the highly connected genes in SVs genes network. CTNNB1, TP53, RB1, ADCY8, MYC, PTK2, PRKCA, TP53, TCF7L2, PAK1, ITPR2, CYP3A4, UGT1A6, CTNNB1, GCK, and FGFR2/3 were among the genes whose alterations could possibly alter a large number of critical biological functions including those which directly infer the cancer mainly the HCC pathways. Moreover, we have also presented the expression (mRNA and protein) (Figure 4D) of some of the potential genes in case of human liver and gallbladder tissues by using the Protein Atlas database (Uhlén et al., 2005, 2017, 2019; Cancer Genome Atlas Research Network, 2008). This study could be an example to apply the integrative approach for a number of complex diseases such cancers, type-2 diabetes, cardiovascular diseases, and neurological disorders (Varambally et al., 2005; Taylor et al., 2010; Van Herle et al., 2012; Zhang et al., 2015; Huwait and Mobashir, 2022).

## Conclusions

According to our findings, only a few genes, such as CLK2, E2F5, CDK5, E2F3, MCM3, PCNA, and CDK4, are highly overexpressed among HCC patients, and the overall expression of all the selected biomarkers appears in more than 60% of the patients, and in terms of co-occurrence, CCNB2, CLK2, CDK4, CDC7, E2F3, PCNA, and MCM3 appear to be the dominantly c Following a temporal analysis, it was discovered that the early stages of HCV infection tend to be more harmful in terms of expression shifting patterns, and that there is no significant change after that, followed by a set of genes that are consistently altered. In contrast to our expression data profile, following 4 days of HCV infection, a group of pathways is always affected. PI3K, cAMP, TGF, TNF, Rap1, NF-kB, Apoptosis, Longevity regulating pathway, signaling pathways regulating pluripotency of stem cells, Cytokine-cytokine receptor interaction, p53 signaling, Wnt signaling, Toll-like receptor signaling, and Hippo signaling pathways are all highly altered pathways in HCC infected with HCV, according to our findings. The majority of these pathways are well-known for their roles in the immune system, infection and inflammation, and human illnesses like cancer. PI3K, cAMP, TGF, TNF, Rap1, NF-kB, Apoptosis, Longevity regulating pathway, signaling pathways regulating pluripotency of stem cells, Cytokine-cytokine receptor interaction, p53 signaling, Wnt signaling, Toll-like receptor signaling, and Hippo signaling pathways are just a few of the most commonly enriched pathways. Most of these pathways are well-known for their functions in the immune system, infection and inflammation, and human diseases such as cancer. According to the networks of genes and pathways based on CNAs, mutations, and SVs, ADCY8, MYC, PTK2, CTNNB1, TP53, RB1, PRKCA, TCF7L2, PAK1, ITPR2, CYP3A4, UGT1A6, GCK, and FGFR2/3 appear to be among the prominent genes.

## Methods

We have selected genome-wide expression and mutational data for HCC with HCV infection and without HCV infection samples. By applying computational approach and integrating experimental data, we have unraveled the critical genes and the pathways which appear to be associated with human HCC. We have selected different datasets and the dataset details are as follows: In GSE63863 (https://www.ncbi.nlm.nih.gov/geo/query/acc.cgi?acc=GSE63863), using the Mass Array EpiTyper, they have looked at a TERT methylation assay that included the UTSS region in 125 matched HCC samples and then analyzed a validation set of 12 matched HCC samples and obtained the TERT gene’s FPKM value to determine the association between TERT promoter methylation status and TERT expression level. In case of GSE14520 (https://www.ncbi.nlm.nih.gov/geo/query/acc.cgi?acc=gse14520), tumors and the associated non-tumor tissues were analyzed independently using a single channel array technology for gene expression profiling. On Affymetrix GeneChip HG-U133A 2.0 arrays, tumor and paired non-tumor samples from 22 patients in cohort 1 and the normal liver pool were analyzed according to the manufacturer’s methodology. An Affymetrix GeneChip Scanner 3000 was used to measure fluorescence intensities, which was controlled by GCOS Affymetrix software. The 96 HT HG-U133A microarray platform was used to process all samples from cohort 2 as well as 42 tumor and non-tumor samples. An Affymetrix GeneChip HT Array Plate Scanner was used to determine the fluorescence intensities, which was controlled by GCOS Affymetrix software. We have also used HCV specific dataset GSE126831 (https://www.ncbi.nlm.nih.gov/geo/query/acc.cgi?acc=GSE126831) where integrated genomic analysis was used to investigate time-resolved HCV infection of hepatocyte-like cells and they discovered pathways relevant for liver disease pathogenesis that have verified in the livers of 216 cirrhotic patients with HCV using differential expression, gene set enrichment analysis, and protein-protein interaction mapping.

In this study, from on previous study, we have collected the genes as biomarkers in case of HCC and studied their clinical relevance and have also studied the publicly available dataset (GSE126831 (Lupberger et al., 2019a)) related to gene expression profiling. In comparison from the previous work, we have applied different approach where we have started our work by mapping the known association (publicly available network database) FunCoup (Alexeyenko and Sonnhammer, 2009), investigated the clinical significance of the overexpression of HCC biomarkers, and finally studied DEGs and the enriched pathways from the gene expression data (obtained from Gene Expression Omnibus). Further, we have utilized the HCC datasets from TCGA database and by using cBioPortal explored all possible mutations, CNAs, and SVs (Koboldt et al., 2012; Werner et al., 2014).

Initially, we have selected the dataset (raw expression dataset) GSE126831 (Lupberger et al., 2019a) for HCC and processed it for normalization and log2 values of all the mapped genes. GSE126831 comprises 63 samples ranging from day 0–10 (temporal samples infected with HCV and mocked samples), with three mocked RNA samples for day 0 and three mocked and three RNA infected with HCV samples for days 1–10. We compared faked samples to HCV infected samples at the respective day of infection for differential gene expression profiling. mRNA profiles of sham or HCV-infected Huh7.5.1dif cells, obtained every day between days 0 and 10 after infection in triplicate. At 7 days after infection, the HCV infection had reached a halt (pi). Unspecific effects cannot be ruled out after day 7 pi.

The paired-end reads from all 63 samples were aligned to the human hg19 UCSC reference using TopHat software for transcriptome profiling at Illumina NextSeq 500 (Homo sapiens) RNA sequencing (v2.0.14). The Cufflinks package’s cuffquant and cuffnorm were used to calculate gene expression levels (FPKM values) (v2.2.1). By creating analytical groups, proteins and transcripts were mapped. Supplementary Files format and content: hg19 Genome build: hg19 The RPKM values for each sample and the results of a differential expression analysis of mapped transcripts are stored in tab-delimited text files. Now, we proceed for our major goal which is to understand the gene expression patterns (Lapointe et al., 2004; Subramanian et al., 2005) and its inferred functions (Subramanian et al., 2005; Mi et al., 2016) and also the impact of HCC biomarker genes. We used MATLAB tools (e.g., mattest) for differential gene expression prediction and statistical analysis, and for pathway analysis, we used the KEGG database (Kanehisa et al., 2007, 2009) and in-house code created for pathway and network research (Bajrai L. et al., 2021; Kamal et al., 2020; Khouja et al., 2022a; Kumar et al., 2020; Warsi et al., 2020). Furthermore, we took all of the HCC samples from the TCGA database and used cBioPortal to look for mutations, CAN, and SV, as well as prepare a list of genes using a threshold cutoff. The CNA threshold was set at 10.0, the mutation threshold at 3.0, and the SV threshold was set at 0.5. As previously stated, this collection of genes has been processed for pathway enrichment analysis. For the GEO datasets, GEO2R was applied for the calculation of *p*-values and fold changes. GEO2R is a web-based tool that allows users to compare two or more groups of Samples in a GEO Series to find genes that are differentially expressed under different experimental settings. The results are supplied as a table of genes ordered by significance, as well as a set of graphic graphs to help visualise differentially expressed genes and assess data set quality (Bajrai L. et al., 2021; Bajrai et al., 2021 L. H.; Eldakhakhny et al., 2021; Khouja et al., 2022b). FunCoup (Reynolds et al., 2010) was used to generate DEGs networks for all of the networks in this study, and cytoscape was utilized to visualize the networks. Protein complexes, protein-protein physical interactions, metabolic, and signaling pathways are among the four types of functional coupling or linkages predicted by FunCoup. MATLAB has been used for the majority of our code and calculations. Cytoscape (Shannon et al., 2003; Skov et al., 2012), network database (PPI), ProgeneV2, and other fundamental tools are among the extra applications and resources used (Krishnamoorthy et al., 2020; Bajrai L. et al., 2021; Bajrai et al., 2021 L. H.; Eldakhakhny et al., 2021; Ahmed et al., 2022; Anwer et al., 2022).

## Data Availability

The original contributions presented in the study are included in the article/Supplementary Material, further inquiries can be directed to the corresponding authors.
